# Neutrophil membrane-mimicking nanodecoys with intrinsic anti-inflammatory properties alleviate sepsis-induced acute liver injury and lethality in a mouse endotoxemia model

**DOI:** 10.1016/j.mtbio.2022.100244

**Published:** 2022-03-16

**Authors:** Yao Xiao, Chao Ren, Gan Chen, Pan Shang, Xiang Song, Guoxing You, Shaoduo Yan, Yongming Yao, Hong Zhou

**Affiliations:** a Institute of Health Service and Transfusion Medicine, Beijing, China; b Translational Medicine Research Center, Fourth Medical Center and Medical Innovation Research Division of the Chinese PLA General Hospital, Beijing, China; c Department of Pulmonary and Critical Care Medicine, Beijing Chaoyang Hospital, Capital Medical University, Beijing, China; d Beijing Institute of Radiation Medicine, Beijing, China

**Keywords:** Sepsis, Neutrophil infiltration, Acute liver injury, Nanovesicles, Inflammatory mediators

## Abstract

Sepsis-induced acute liver injury often develops in the early stages of sepsis and can exacerbate the pathology by contributing to multiple organ dysfunction and increasing lethality. No specific therapies for sepsis-induced liver injury are currently available; therefore, effective countermeasures are urgently needed. Considering the crucial role of neutrophils in sepsis-induced liver injury, herein, neutrophil membrane-mimicking nanodecoys (NM) were explored as a biomimetic nanomedicine for the treatment of sepsis-associated liver injury. NM administration exhibited excellent biocompatibility and dramatically decreased the plasma levels of inflammatory cytokines and liver injury biomarkers, including aspartate aminotransferase, alanine aminotransferase, and direct bilirubin, in a sepsis mouse model. NM treatment also reduced hepatic malondialdehyde content, myeloperoxidase activity, and histological injury, and ultimately improved survival in the septic mice. Further *in vitro* studies showed that NM treatment neutralized the neutrophil chemokines and inflammatory mediators and directly mitigated neutrophil chemotaxis and adhesion. Additionally, NM also markedly weakened lipopolysaccharide-induced reactive oxygen species generation, cyclooxygenase-2 expression, nitric oxide secretion, and subsequent hepatocyte injury. Thus, this study provides a promising therapeutic strategy for the management of sepsis-induced acute liver injury.

## Graphical abstract

**Figure:**
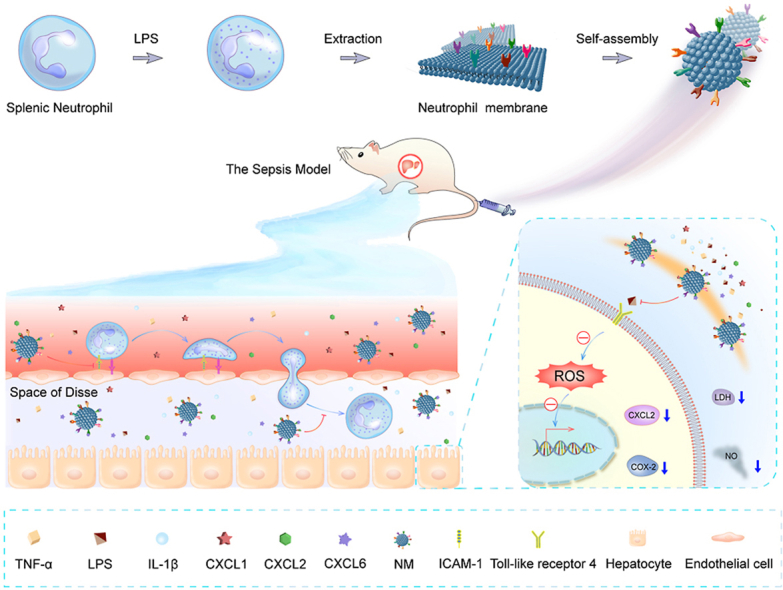

## 1 Introduction

Sepsis, a life-threatening syndrome associated with excessive inflammation and consequent organ dysfunction triggered by trauma or overwhelming infection [1,2], is the most common cause of death in intensive care units [3]. During the development of sepsis, acute liver injury often occurs at the early stage [4] and is directly associated with a poor prognosis in clinical investigations [5,6]. Therefore, it is critical to prevent sepsis-induced acute liver injury at the early stage to improve the outcome of septic patients. No specific therapies for sepsis-induced liver injury are currently available [7], therefore, the development of effective alternative therapeutic strategies for sepsis-induced acute liver injury is urgently needed.

The precise pathogenesis of sepsis-associated acute liver injury remains unknown. However, accumulating evidence implicates that neutrophil infiltration and overwhelming proinflammatory cytokine production play major roles in this process [5,8]. Notably, neutrophils are recruited to the liver in response to inflammatory mediators and chemokines, wherein they adhere to inflamed endothelial cells and migrate to the parenchyma to eliminate the pathogens [9]. Activated neutrophils also contribute to tissue injury by directly releasing toxic effectors and exaggerating the inflammatory response simultaneously [10]. In addition, various proinflammatory cytokines, including tumor necrosis factor (TNF)-α, interleukin (IL)-1β, and IL-6, can trigger and amplify liver destruction and acute liver injury [11]. Therefore, therapeutic strategies aimed at simultaneously alleviating neutrophil infiltration and blocking the action of proinflammatory cytokines could be beneficial in the treatment of sepsis-induced acute liver injury.

Recently, cell membrane-camouflaged nanoparticles have emerged as a promising therapeutic platform for broad applications [[12], [13], [14]]. In particular, neutrophil membrane-camouflaged nanoparticles were used for inflammation-targeted drug delivery [[15], [16], [17], [18]]. Considering the pivotal role of neutrophils in the pathogenesis of sepsis-associated liver injury, we hypothesized that nanovesicles derived from neutrophils might be an effective therapeutic for sepsis-induced liver injury by regulating neutrophil infiltration and neutralizing inflammatory mediators.

In this study, we developed neutrophil membrane-mimicking nanodecoys (NM) and systematically explored the therapeutic potential for sepsis-induced liver injury. Red cell membrane-mimicking nanovesicles (RM) were used as a control to exclude the potential effect of non-specific binding of inflammatory factors and chemokines on the results. We indicated that NM constructed using the simple extrusion method could reduce hepatic neutrophil infiltration, and subsequent liver injury by neutralizing the neutrophil chemokines and inflammatory mediators in a septic mouse model. Furthermore, NM also markedly weakened lipopolysaccharide (LPS)-induced reactive oxygen species (ROS) generation, cyclooxygenase-2 (COX-2) expression, nitric oxide (NO) secretion, and subsequent hepatocyte injury.

## 2 Materials and methods

### 2.1 Materials

Pierce™ BCA protein assay kit was obtained from Thermo Fisher Scientific (CA, USA). LPS (*Escherichia coli* 0111:B5), 2ʹ-7ʹ-dichlorofluorescein diacetate (DCFH-DA), and dimethyl sulfoxide (DMSO) were purchased from Sigma-Aldrich (MO, USA), and nucblue live cell stain readyprobes were purchased from invitrogen (CA, USA).

### 2.2 Cell membrane extraction

The neutrophils were extracted from Balb/c mouse spleens using sucrose density gradient centrifugation, according to the manufacturer's instructions (TBD Science, China). After LPS stimulation (2 ​mg/mL) for 4 ​h, the neutrophil membrane was obtained using a membrane protein extraction kit (Beyotime, China). Briefly, after washing with phosphate buffered saline (PBS), the LPS-stimulated neutrophils were collected by centrifugation (700×*g*, 4 ​°C) and subsequently suspended by the membrane protein extraction reagent A with protease inhibitor cocktail (MedChem Express, NJ, USA) in an ice-water bath. The mixture was then freeze-thawed repeatedly for three cycles after centrifugation (700×*g*, 10 ​min, 4 ​°C), and the supernatant was further centrifuged (14,000×*g*, 30 ​min, 4 ​°C) for neutrophil membrane isolation. Finally, the collected neutrophil membrane was stored at −80 ​°C for further use.

The heparinized whole blood from male Balb/c mice was centrifuged (800×*g*, 5 ​min, 4 ​°C) to separate red blood cells (RBC). The obtained RBCs were then washed using ice-cold PBS and suspended in hypotonic 0.25 ​× ​PBS (isotonic PBS diluted 1:3 with deionized water) containing protease inhibitor cocktail in an ice bath for 4 ​h. The mixture was centrifuged (14,000×*g*, 30 ​min, 4 ​°C) and repeatedly washed to remove hemoglobin. The RBC membrane was collected and stored at −80 ​°C for further assay.

### 2.3 Preparation and characterization of NM and RM

The thawed neutrophil membrane and RBC membrane were re-dispersed, quantified, and sonicated for 5 ​min at a frequency of 40 ​kHz and power of 100 ​W in a capped glass vial. The resulting suspensions were subsequently extruded using an Avanti mini extruder (Avanti Polar Lipids, AL, USA) through 200 ​nm polycarbonate porous membranes 11 times to obtain the NM and RM, respectively.

The morphology of nanovesicles was observed using a FEI Talos F200C transmission electron microscope (TEM) instrument (200 ​kV) equipped with an SC 1000 CCD camera (Thermo Fisher Scientific, NY, USA). The particle size distribution and zeta potential were determined by a Zetasizer Nano-ZS (Malvern, UK).

Protein characterization was performed by sodium dodecyl sulfate poly-acrylamide gel electrophoresis (SDS-PAGE). After electrophoresis, the obtained gels were stained with Coomassie blue staining solution, then washed three times with Coomassie Blue eluent and distilled water. The gel was finally recorded by an imaging system (Bio-Rad, CA, USA).

### 2.4 Pharmacokinetics and biodistribution *in vivo*

After intravenously administration of DiD-labeled NM (40 ​mg/kg), 33 Balb/c mice (male, 6–8 weeks) were divided into 11 groups. Approximately 20 ​μL of venous blood was collected at 1 and 10 ​min, and 1, 2, 4, 8, 12, 24, 48, 72, and 96 ​h after administration. Blood was collected from one group of mice at each time point. The obtained blood was then diluted using 180 ​μL normal saline for fluorescence measurement.

For tissue biodistribution study, the major organs, including liver, kidneys, spleen, lungs, and heart, were collected at desired time points (10 ​min, and 4, 8, and 24 ​h) and homogenized in ice-cold normal saline. The fluorescence intensities of tissue homogenate were detected using a microplate reader (Molecular Devices, USA) equipped with 644 ​nm excitation and 663 ​nm emission filters after dilution to the same concentration.

### 2.5 Animal care and mouse endotoxemia model

All animal experiments conformed to the Guide for the Care and Use of Laboratory Animals and approved by the Institutional Animal Care and Use Committee of the Academy of Military Medical Sciences. The Balb/c mice (Male, 6–8 weeks) were acquired from Vital River (Beijing, China) and used for experiments after a minimum of 3 days of acclimation, following standard laboratory procedures.

To evaluate the therapeutic effect, mice were randomized into control or experimental groups according to the different treatments, namely, the normal control group (CK) and the blank NM-treated group (40 ​mg/kg) as control groups, and experimental groups were intravenously administered with NM (40 ​mg/kg), RM (40 ​mg/kg), or an equal volume of PBS vehicle after 30 ​min of LPS intraperitoneal administration (10 ​mg/kg). Mice were sacrificed 8 ​h after LPS administration, and the plasma and livers were then immediately collected and stored for further analysis. The dosage of 40 ​mg/kg was in reference to a previous study [19], however, it was subjected to modification.

For the survival experiments, 85 mice were randomized into control or sepsis groups. The sepsis groups were intravenously administered with NM (40 ​mg/kg, n ​= ​25), RM (40 ​mg/kg, n ​= ​25) or an equal volume of PBS vehicle (n ​= ​25) after a lethal dose of LPS via intraperitoneal administration (40 ​mg/kg), respectively. The normal mice were used as the control group (n ​= ​10). The survival of mice was observed for 96 ​h.

### 2.6 Plasma biochemistry

Plasma biochemistry was analyzed using a blood chemistry analyzer (MNCHIP, Tianjin, China).

### 2.7 Measurement of chemokines and inflammatory mediators

The IL-1β, IL-6, TNF-α, CXC-Chemokine Ligand 1 (CXCL1), CXCL2, and CXCL6 levels were determined using enzyme-linked immunosorbent assay (ELISA) kits (PeproTech, NJ, USA) as previously reported [20]. The LPS content was measured using ELISA kit (Nanjing Jiancheng Bioengineering Institute, Nanjing, China) according to the manufacturer's instructions.

### 2.8 Quantitative real-time polymerase chain reaction

Total RNA was extracted from livers using the Ultrapure RNA Kit (CoWin Biosciences, Beijing, China), followed by reverse transcription using the PrimeScript™ RT Master Mix (TakaRa, Dalian, China). Quantitative real-time PCR was performed using SYBR Green Master Mix (Toyobo, Osaka, Japan) according to the manufacturer's protocol. 18s was used as the internal reference for the expression of IL-6, IL-1β, and TNF-α. Sequences of the primers employed were provided in Table 1S.

### 2.9 Neutrophil infiltration and lipid peroxidation in the liver

The livers were homogenized in ice-cold normal saline and centrifuged (1000×*g*, 6 ​min, 4 ​°C), and the supernatants were analyzed for the myeloperoxidase (MPO) activity and malondialdehyde (MDA) content (Nanjing Jiancheng Biological Institute, Nanjing, China) according to the manufacturer's instructions as previously described [21]. Total protein levels of homogenates were detected using a BCA protein assay kit.

### 2.10 Histological analysis and terminal deoxynucleotidyl transferase dUTP nick end labeling

The paraformaldehyde-fixed liver tissues were dehydrated, embedded, and subsequently sectioned into 4 ​μm thick slices. The slices were then subjected to staining with hematoxylin and eosin. The severity of liver damage in the hepatic lobule and portal area was assessed for inflammatory infiltration, cell swelling, and tissue architecture disruption in a blinded fashion using light microscopy and scored on a 4-point scale (0, none; 1, slight; 2, moderate; 3, severe).

For TUNEL staining, the fixed liver tissues were stained for DNA stand breaks by TUNEL assay using the In Situ Cell Death Detection Kit (Roche Diagnostics, Mannheim, Germany) according to the manufacturer's instruction.

### 2.11 Immunohistochemistry and immunofluorescence

Immunohistochemical staining was performed to measure the expression of lymphocyte antigen 6 complex locus G6D (Ly6G) and Bax in the liver sections. After deparaffinization, rehydration, antigen retrieval, and endogenous enzyme blocking, the sections were incubated with a rabbit anti-Bax antibody (1:50, Cell Signaling Technology, MA, USA) or a rabbit anti-Ly6G antibody (1:50, Thermo Fisher Scientific, NY, USA). The sections were then washed and incubated with HPR-labeled secondary anti-rabbit antibody. The immunostaining signal was subsequently visualized using Diaminobenzidine (DAB).

For immunofluorescent staining of intercellular adhesion molecule-1 (ICAM-1), F4/80, and inducible nitric oxide synthase (iNOS), after incubated with a rabbit anti-ICAM-1 antibody (1:50, Proteintech, IL, USA), rat anti-F4/80 antibody (1:50, Thermo Fisher Scientific, NY, USA), or a rabbit anti-iNOS antibody (1:200, Proteintech, IL, USA), the liver sections were washed and incubated with the HPR-labeled secondary antibodies. The signal was amplified using Cy3-conjugated tyramide signal amplification (TSA) fluorescence systems (Servicebio, China). For immunofluorescent analysis of COX-2 and iNOS expression, the cell samples were incubated with a rabbit anti-COX-2 antibody (1:50, Proteintech, IL, USA) or a rabbit anti-iNOS antibody (1:200), washed, and visualized using Alexa Fluor 488 conjugated goat anti-rabbit IgG or Alexa Fluor 568 conjugated goat anti-rabbit IgG (Thermo Fisher Scientific, CA, USA). All the samples were finally stained with 4′,6-diamidino-2-phenylindole (DAPI) for nuclear staining and imaged using a fluorescence microscope.

### 2.12 Western blot analysis

Samples were extracted and quantified following denaturation, and equivalent them were loaded into a 10% polyacrylamide gel, then transferred onto polyvinylidene fluoride (PVDF) membranes. After blocking, the PVDF membranes were incubated with primary anti-body. Finally, the PVDF membranes were further incubated with horseradish peroxidase (HRP) conjugated secondary antibody (Thermo Fisher, CA, USA), and the blots were developed by a west Pico PLUS chemiluminescent substrate kit (Thermo Fisher, CA, USA). The following antibodies were used: anti-TNFR1 (Sino Biological, Beijing, China), Ly6G (Thermo Fisher Scientific, NY, USA), COX-2 (Proteintech, IL, USA), and β-actin antibody (Sigma-Aldrich, MO, USA).

### 2.13 Serum amyloid A-Luc mouse model construction and bioluminescence imaging

The Serum Amyloid A (SAA)-Luc reporter mouse model was established as previously described using hydrodynamic gene delivery technology [19,21]. Therefore, the plasmid pattB-SAA-Luc in saline was administrated by rapid hydrodynamic tail vein injection within 5 ​s at a dosage of 0.1 ​mL/g body weight. Two weeks later, the model mice were subjected to LPS administration, and the effects of NM and RM on serum Amyloid A (SAA) activation were evaluated by an IVIS imaging system (Xenogen, Alameda, CA, USA) after D-fluorescein intraperitoneal injection (150 ​mg/kg).

### 2.14 Inflammatory mediators and chemokine binding studies

To determine the binding capacities of NM and RM with LPS, the NM and RM in PBS containing 10% FBS with varying concentrations (10, 5, 2.5, and 1.25 ​mg/mL) were mixed with an equal volume of LPS (400 ​ng/mL) in PBS. The mixtures were then incubated at 37 ​°C for 30 ​min, followed by centrifugation (14,000×*g*, 45 ​min). The concentration of LPS in the supernatant was quantified using an ELISA kit.

To assess the binding capacities of NM and RM with IL-1β, TNF-α, CXCL1, CXCL2, CXCL6, and SAA, the NM and RM in PBS with varying concentrations (8, 4, 2, and 1 ​mg/mL) were mixed with equal volume of IL-1β (2000 ​ng/mL), TNF-α (600 ​ng/mL), CXCL1 (600 ​pg/mL), CXCL2 (1200 ​pg/mL), CXCL6 (1600 ​pg/mL), or SAA (400 ​ng/mL) in PBS. The mixtures were incubated at 37 ​°C for 30 ​min, followed by centrifugation (14,000×*g*, 45 ​min). The concentrations of different cytokines in the supernatant were determined using ELISA methods.

### 2.15 Transwell assay

The peripheral blood neutrophils were isolated and suspended in RPMI 1640 medium (1 ​× ​10^5^ ​cells/mL). A 100 ​μL sample of neutrophil suspension was added to the upper chamber with a 3 ​μm pore polyester membrane (Corning, NY, USA). The NM or RM in RPMI 1640 medium containing 10% fetal bovine serum (FBS) was added to the bottom chamber. After incubation for 30 ​min at 37 ​°C and 5% CO_2_, the medium in the bottom chamber was collected for counting the migrating neutrophils using a hemocytometer.

### 2.16 Cell culture, treatment, and injury evaluation

The NCTC1469 murine liver cells and C166 mouse endothelial cells were obtained from China Center for Type Culture Collection and cultured in a DMEM medium (Life Technologies, CA, USA) supplemented with 10% FBS (Life Technologies, CA, USA) and 1% penicillin-streptomycin solution (Thermo Fisher Scientific, MA, USA) at 37 ​°C in a humidified atmosphere of 5% CO_2_.

The neutralization efficacy of NM and RM with LPS was further investigated by the LPS-induced liver cell injury model. A 500 ​μL of NM (10 ​mg/mL) or RM (10 ​mg/mL) solution mixed with 500 ​μL of LPS (200 ​ng/mL) in a cell culture medium, after incubation for 30 ​min at 37 ​°C, the mixture was centrifuged (14,000×*g*, 45 ​min). The supernatant was collected to culture NCTC1469 ​cells for 24 ​h, and the cell viability was measured using the Cell Counting Kit-8 (CCK-8) assay (LABLEAD, Beijing, China).

For the intracellular reactive oxygen species (ROS) measurement, after LPS treatment for 24 ​h, the NCTC1469 ​cells were incubated with DCFH-DA (10 ​μM) for 30 ​min and washed twice by PBS, followed by fluorescence intensity quantification by a microplate reader (Molecular Devices, USA) as previously reported [22]. Lactate dehydrogenase (LDH) activity in the cell culture supernatant was analyzed using the Cytotoxicity LDH Assay Kit (Dojindo Laboratories, Kumamoto, Japan) according to the manufacturer's instruction. The nitric oxide (NO) content in cell culture medium was detected using the commercial Nitric Oxide Assay Kit (Abnova, Taipei, China).

### 2.17 Neutrophil-endothelial cell adhesion

To assess neutrophil-endothelial cell adhesion, A 500 ​μL of NM (10 ​mg/mL) or RM (10 ​mg/mL) solution was mixed with 500 ​μL of LPS (200 ​ng/mL) in cell culture medium, the mixture was centrifuged (14,000×*g*, 45 ​min) after incubation for 30 ​min at 37 ​°C, and the supernatant was collected to culture C166 ​cells for 12 ​h. After washed three times by PBS, the nucblue live cell stain readyprobes reagent labeled neutrophils in a fresh medium (5 ​× ​10^6^ ​cells/mL) were added and incubated in a fresh medium for 30 ​min under normal culture conditions. Next, the cells were quantified by a microplate reader and imaged using a confocal microscopy (Nikon A1, Tokyo, Japan) after washing them three times with PBS.

### 2.18 Statistical analysis

All data are presented as mean ​± ​standard error of mean (SEM). Statistical differences between groups were performed by a one-way analysis of variance (ANOVA), followed by Student Newman-Keuls (SNK) multiple comparisons when the homogeneity and normality of the variance assumptions were satisfied. Otherwise, ANOVA, followed by the SNK multiple range test was used. The unpaired student's t-test was used for the assessment of statistically significant differences between the two groups in inflammatory mediators and chemokine binding studies. Survival data were analyzed by the log-rank test. *P* values ​< ​0.05 were considered significant.

## 3 Results

### 3.1 Construction and characterization of nanodecoys

**Fig. 1:**
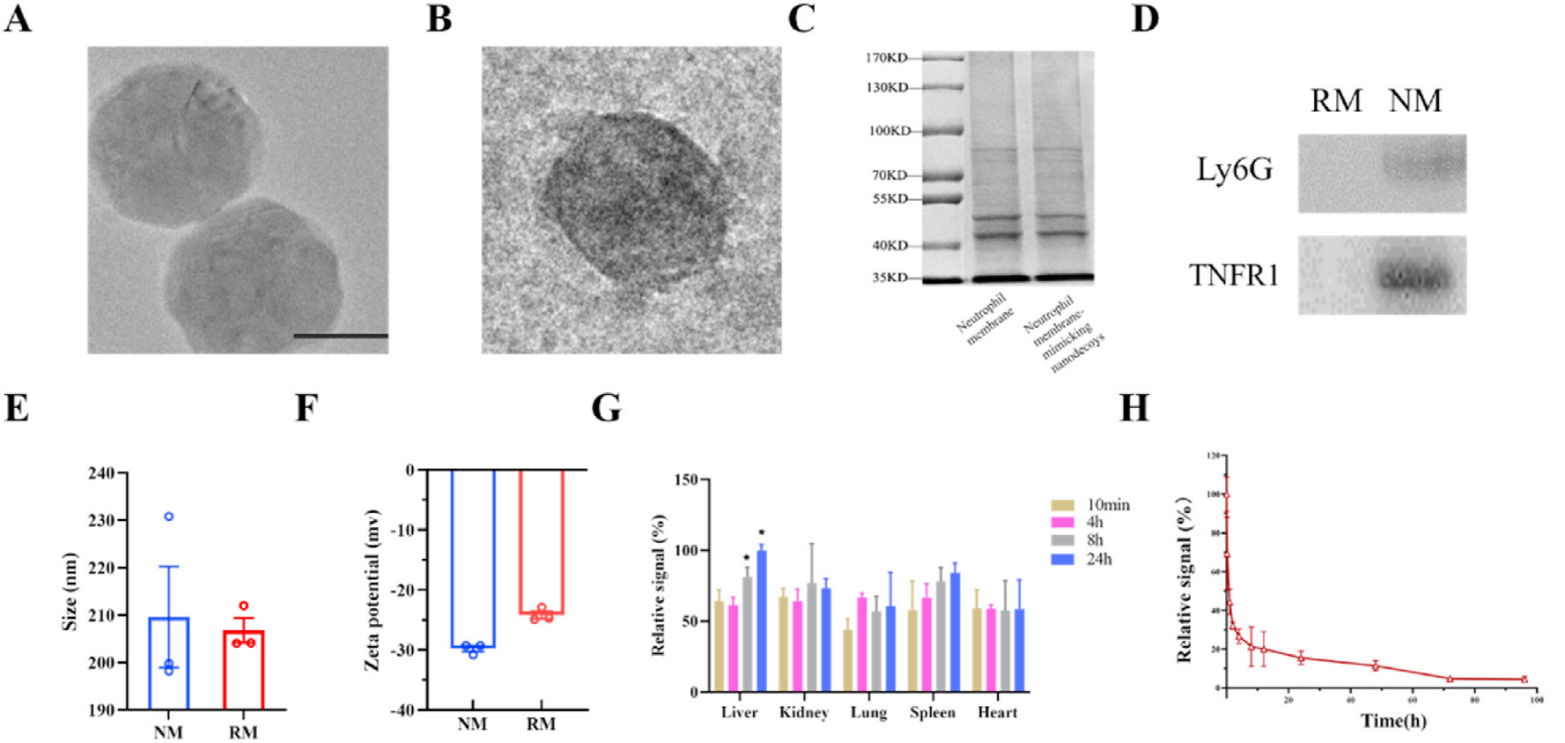
Construction and characterization of neutrophil membrane-mimicking nanodecoys (NM) and red cell membrane-mimicking nanovesicles (RM). (A) and (B) The Cryo-electron micrograph of NM and RM (scale bar, 100 ​nm). (C) Protein profiles of the neutrophil membrane and NMs assessed by sodium dodecyl sulfate poly-acrylamide gel electrophoresis (SDS-PAGE). (D) Western blotting analysis of lymphocyte antigen 6 complex locus G6D (Ly6G) and tumor necrosis factor receptor −1 (TNFR1) in RM and NM. (E) and (F) Average diameter size and zeta potential of NM and RM. (G) The biodistribution of NM in major organs (n ​= ​3, statistical differences between groups were performed by a one-way ANOVA) and fluorescence intensity of liver at 24 ​h was set as 100%. ∗*p* ​< ​0.05 compared with the fluorescence intensity of liver at 10 ​min. (H) Blood circulation time of NM (n ​= ​3). (For interpretation of the references to color in this figure legend, the reader is referred to the Web version of this article.)

The NM and RM were prepared by sonication and the extrusion method. Cryo-electron microscopy showed that NM and RM exhibited a spherical shape with good monodispersity and an average size of 180 ​nm (Fig. 1A and B). Additionally, SDS-PAGE of NM with neutrophil membrane was performed in parallel (Fig. 1C).

The tumor necrosis factor receptor −1 (TNFR1) expressed on cell membrane can function as TNF-binding proteins that decrease TNF-α activity, and Ly6G is a highly specific marker for neutrophils, Thus the expression level of TNFR1 and Ly6G is detected in this study. The results of western blot showed that TNFR1 and Ly6G expression levels of NM were significantly higher than that of RM (Fig. 1D). Dynamic light scattering (DLS) measurements indicated that the average hydrodynamic diameters were 209.6 ​± ​15.0 ​nm for NM and 206.7 ​± ​3.7 ​nm for RM. DLS results showed a sphere of hydration around the nanoparticles, which was larger than the TEM size. The surface zeta potential of NM and RM were −29.8 ​± ​0.7 and −24.2 ​± ​0.9 ​mV, respectively (Fig. 1E and F). The results showed that the NM and RM have good stability and suggested that the endogenous membrane proteins of the neutrophil membrane were preserved on NM.

The DiD dye was used to investigate the pharmacokinetics of NM by labeling nanodecoys. The biodistribution results showed that NM contents in the liver and spleen were increased with the prolongation of the time, which indicated that the reticuloendothelial system causes the NM uptake in the liver and spleen over time (Fig. 1G). After intravenous administration, NM showed 21.3% and 15.5% retention in the plasma at 8 and 24 ​h, respectively (Fig. 1H).

### 3.2 NM *reduces histological injury and prolongs the survival of septic mice*

**Fig. 2:**
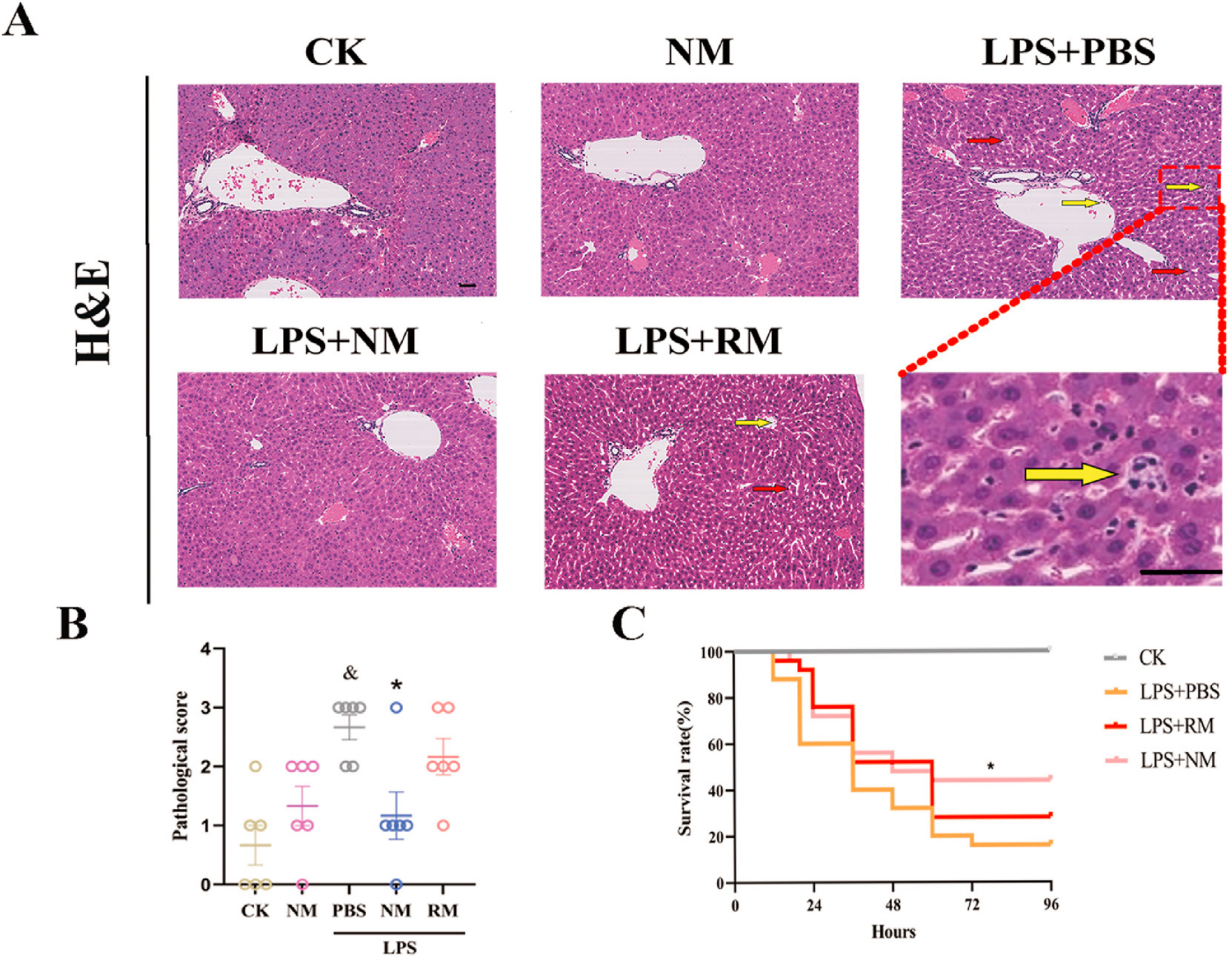
Representative images show the effect of neutrophil membrane-mimicking nanodecoys (NM) and red cell membrane-mimicking nanovesicles (RM) treatment on histological injury (A) (Yellow arrows indicate infiltrating inflammatory cells, and red arrows indicate tissue architecture disruption, Scale bar, 20 ​μm) and histologic liver injury scores (B). (C) Survival rates of sepsis mice over 96 ​h following NM and RM administration. ^&^*p* ​< ​0.05 compared with the CK group, ∗*p* ​< ​0.05 compared with the LPS ​+ ​PBS group. Statistical differences between groups were analyzed by the log-rank test. (For interpretation of the references to color in this figure legend, the reader is referred to the Web version of this article.)

The hepatic histological injury was evaluated. As shown in Fig. 2A and B, there was remarkable liver injury, including inflammatory cell infiltration, tissue architecture disruption, and cell swelling, all of which were significantly attenuated by treatment with NM, while RM administration showed non-significant improvement in the pathological injury of the liver.

To further validate the therapeutic potential of NM for sepsis, the impact of NM treatment on the survival of lethal septic mice was evaluated. The lethal doses of the LPS challenge resulted in the death of mice, and a single dose of NM treatment significantly improved the 96-h survival rate of sepsis model mice from 16% to 44% (*P* ​< ​0.05, Fig. 2C), while RM exhibited non-significant improvement for 96-h survival rate of sepsis model mice.

### 3.3 NM *improves liver injury biomarkers and decreases plasma inflammatory cytokines*

**Fig. 3:**
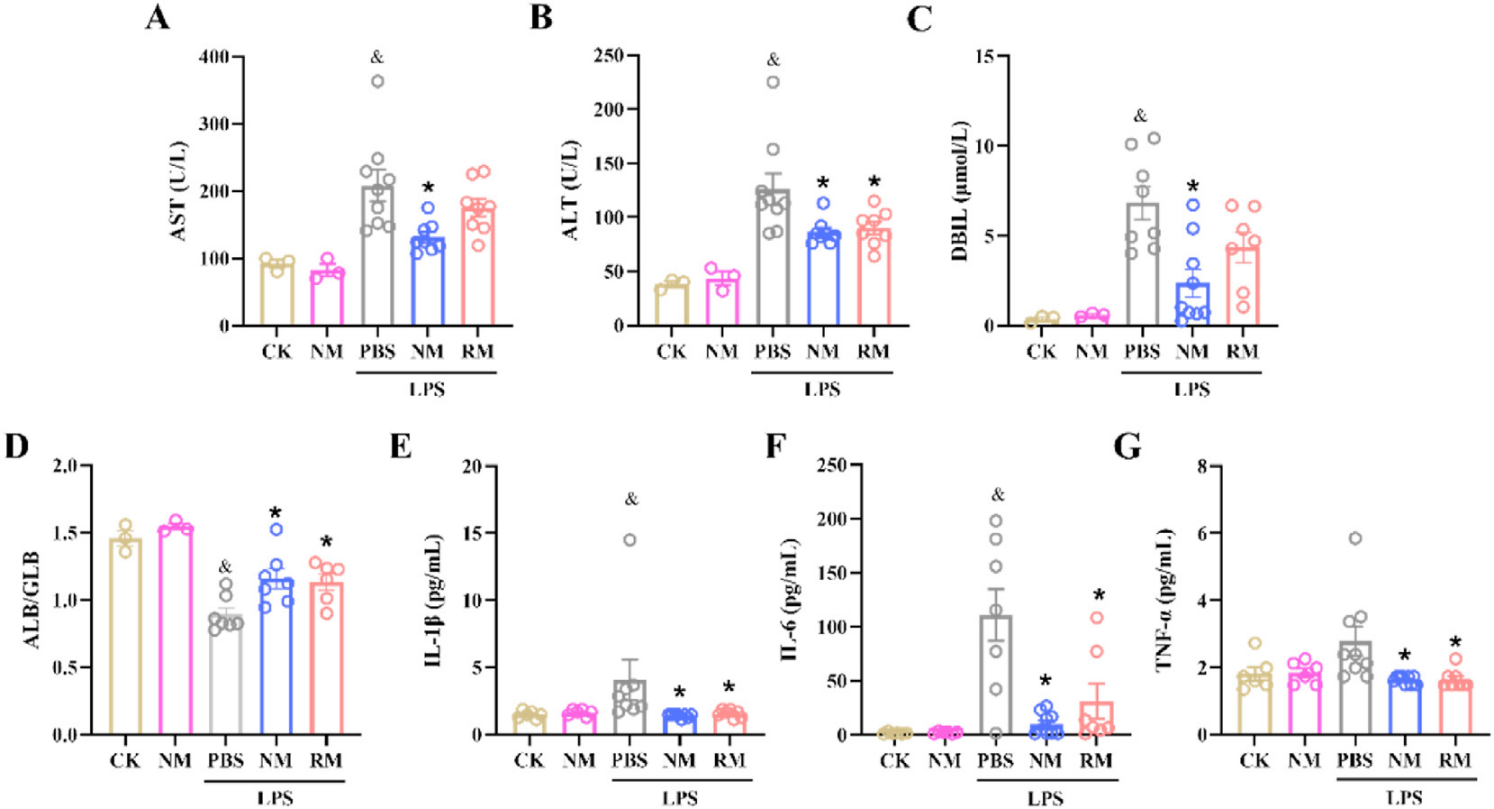
Biochemical indices in plasma and liver tissues. The levels of aspartate aminotransferase (AST) (A), alanine aminotransferase (ALT) (B), direct bilirubin (DBIL) (C), albumin (ALB)/globulin (GLB) ratio (D), interleukin (IL)-1β (E), IL-6 (F), and tumor necrosis factor (TNF)-α (G) in the plasma 8 ​h after LPS administration. ^&^*p* ​< ​0.05 compared with the CK group; ∗*p* ​< ​0.05 compared with the LPS ​+ ​PBS group. Statistical differences between groups were performed by a one-way ANOVA for Fig. 3A, B, D, F, and G. Statistical differences between groups were performed by SNK multiple range test for Fig. 3C and E.

The levels of ALT, AST, DBIL, and the ratio of ALB to GLB in the plasma are used as biomarkers to reflect acute liver injury. The elevated levels of AST, ALT, and DBIL in plasma indicate liver injury [[23], [24], [25]], while the ratio of ALB to GLB in plasma are decreased when there is liver injury [26,27]. As shown in Fig. 3, LPS administration significantly elevated the levels of AST, ALT, and DBIL, and decreased the ratio of ALB to GLB compared to the normal controls, which indicated liver dysfunction in the sepsis group. NM administration significantly decreased the plasma contents of AST, ALT, and DBIL (Fig. 3A, B, and C), while increasing the ratio of ALB to GLB compared to the sepsis control (Fig. 3D). Additionally, RM administration revealed a significant suppression on plasma contents of ALT, and an increase in the ratio of ALB to GLB compared to the sepsis mice.

In comparison to the normal controls, significantly higher plasma contents of IL-1β and IL-6 were found in the septic mice treated with PBS. NM administration significantly diminished the plasma levels of IL-1β, TNF-α, and IL-6 compared to the sepsis control (Fig. 3E, F, and G). Similarly, RM administration significantly decreased the plasma levels of IL-1β, IL-6, and TNF-α.

For *in vivo* therapeutic applications, the biocompatibility of potential therapeutics needs to be evaluated. NM administration had no significant effect on the liver injury biomarkers and inflammatory cytokines in the plasma compared to the normal controls (*P* ​> ​0.05), which suggested that NM exerted no acute toxicity at the used dosage.

### 3.4 NM *alleviates hepati****c****inflammatory cytokines mRNA expression, lipid peroxidation and neutrophil infiltration in the liver*

**Fig. 4:**
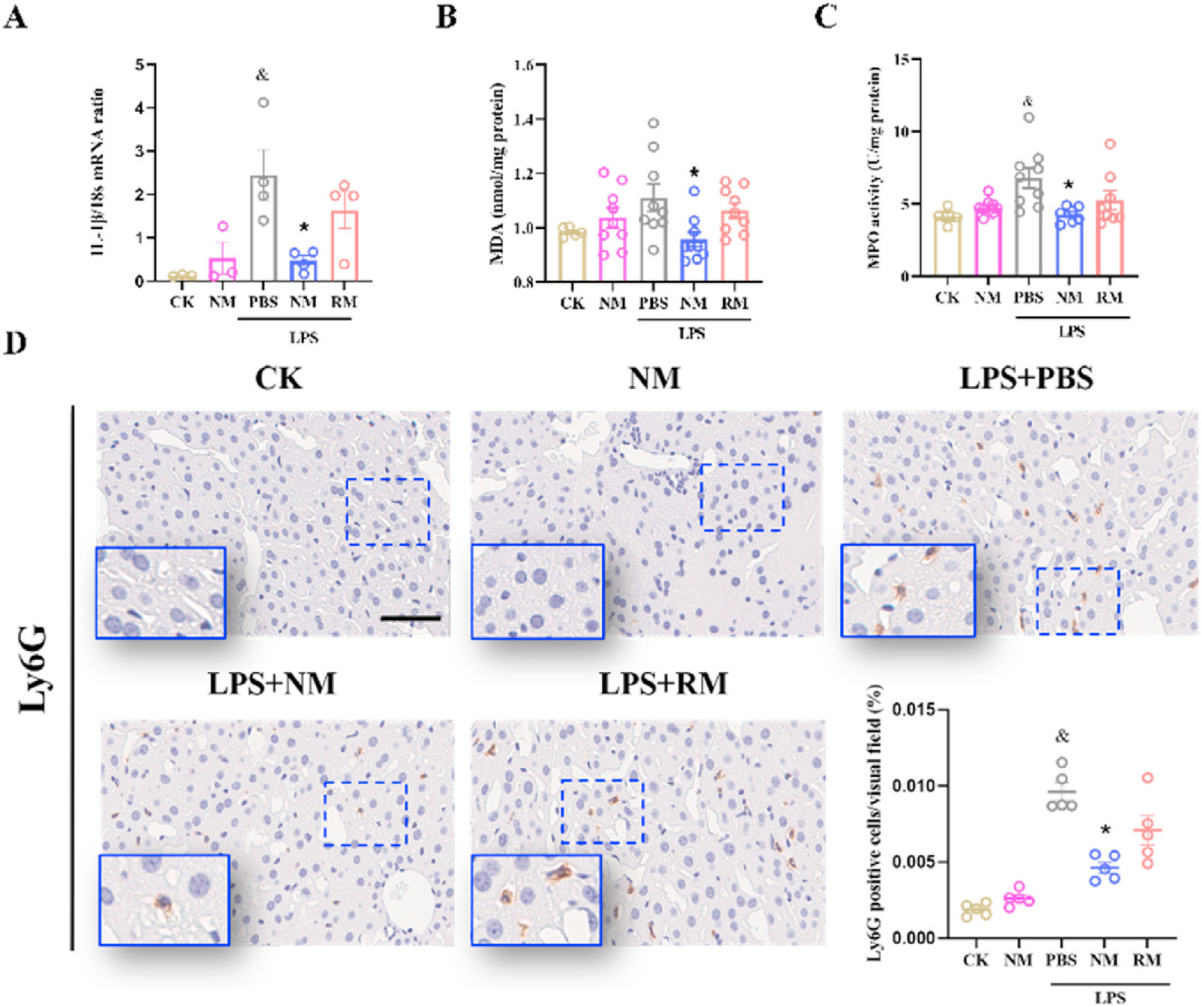
Hepatic interleukin (IL) -1β mRNA expression (A), malondialdehyde (MDA) content (B), and the myeloperoxidase (MPO) activity (C) in the liver. (D) Representative images and quantitative analysis show the effect of neutrophil membrane-mimicking nanodecoys (NM) and red cell membrane-mimicking nanovesicles (RM) treatment on Ly6G protein expression levels in the liver (Scale bar, 50 ​μm). ^&^*p* ​< ​0.05 compared with the CK group; ∗*p* ​< ​0.05 compared with the LPS ​+ ​PBS group. Statistical differences between groups were performed by a one-way ANOVA. (For interpretation of the references to color in this figure legend, the reader is referred to the Web version of this article.)

As shown in Fig. 4A, there were significantly increase in hepatic IL-1β mRNA expression in septic model mice compared with normal control mice. NM treatment significantly reduced IL-1β mRNA expression compared to the sepsis group, whereas RM administration showed a non-significant suppression (*P* ​> ​0.05). For IL-6 and TNF-α mRNA expression, there were significantly increase in hepatic IL-6 and TNF-α mRNA expression in septic model mice compared with normal control mice, both NM and RM exhibited non-significant inhibitory effects (Fig. S1).

Hepatic lipid peroxidation is measured by quantifying the MDA content in the liver [19]. The mice with NM administration showed a significantly reduced MDA content compared to the sepsis group (Fig. 4B). However, the RM at an equal amount as NM did not show a significant inhibitory impact on MDA content.

The MPO activity is used as a quantitative assessment of neutrophil infiltration [28]. As shown in Fig. 4C, there was a significantly higher hepatic MPO activity in septic model mice compared with normal control mice. The mice under NM treatment revealed a significantly reduced MPO activity compared to the sepsis group, whereas the mice with RM administration showed a slight, non-significant suppression (*P* ​> ​0.05). In addition, neutrophil infiltration was verified by immunohistochemical analysis of Ly6G, which is also considered a reliable marker to identify neutrophils [29]. Immunohistochemical staining indicated that significantly increased levels of neutrophils were detected in the sepsis model mice compared with mice in the control group. NM-treated mice showed a significant decrease in neutrophil levels in the liver (Fig. 4D).

### 3.5 NM *reduces apoptosis and macrophage activation in the liver*

**Fig. 5:**
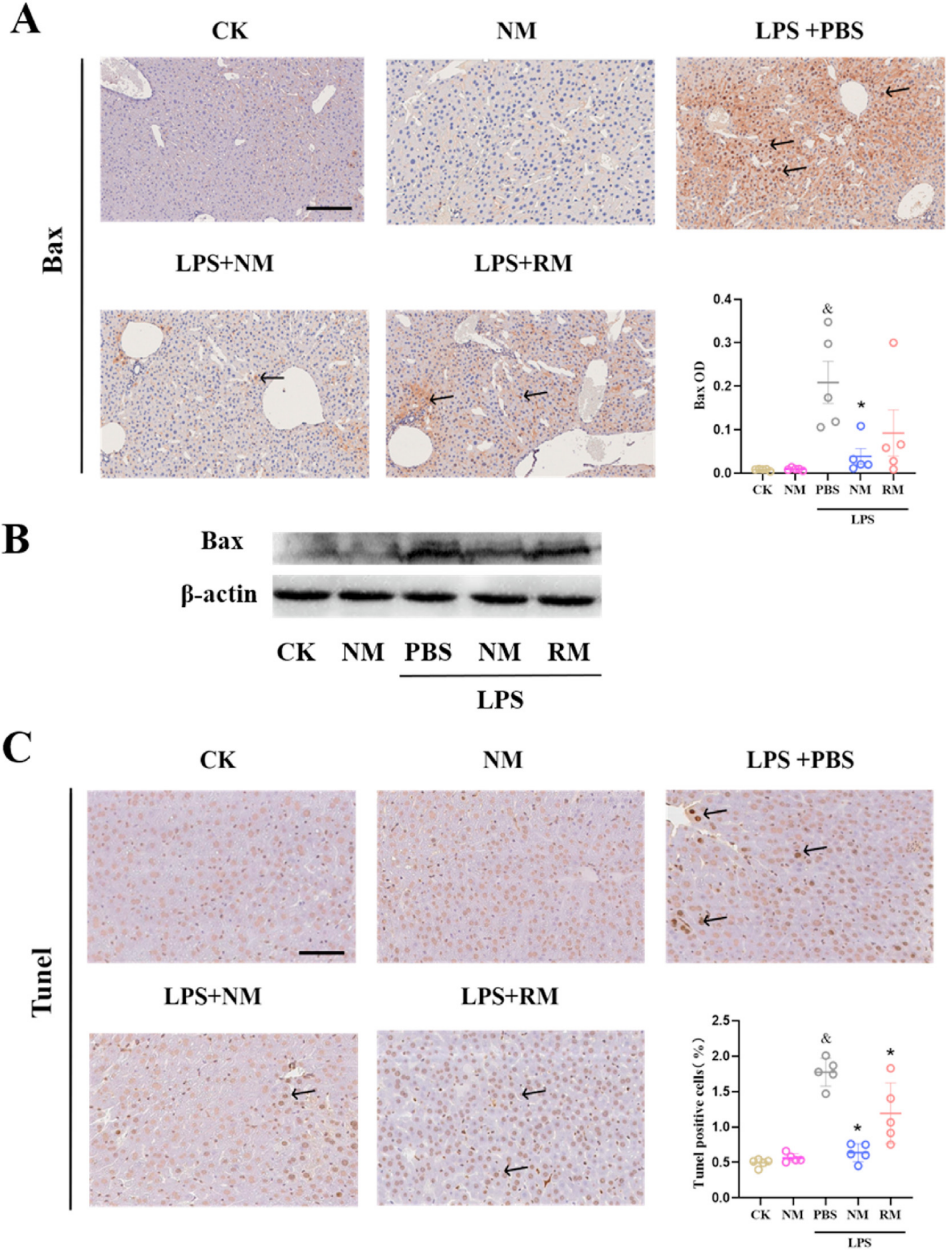
Representative images show the effect of neutrophil membrane-mimicking nanodecoys (NM) and red cell membrane-mimicking nanovesicles (RM) treatment on protein expression levels of Bax (A, B) and apoptosis (C) in the liver (Scale bar, 50 ​μm). ^&^*p* ​< ​0.05 compared with the CK group, ∗*p* ​< ​0.05 compared with the LPS ​+ ​PBS group. Statistical differences between groups were performed by a one-way ANOVA. (For interpretation of the references to color in this figure legend, the reader is referred to the Web version of this article.)

Bax, one of the pro-apoptotic BCL-2 gene family members, plays a key role in the apoptotic pathway [30]. As shown in Fig. 5A, LPS administration significantly enhanced Bax expression, while NM treatment largely prevented upregulated Bax expression induced by LPS administration, and RM treatment showed non-significant suppression of Bax expression. The results of western blot also verified the above results (Fig. 5B). The terminal deoxynucleotidyl TUNEL staining was further used to investigate the apoptosis of the liver cells. There was a significant increase in TUNEL-positive cells in the sepsis model group compared with the control group, however, this effect was remarkably mitigated by the NM treatment. The RM treatment showed a relatively weak suppression of TUNEL-positive cells. Additionally, NM administration did not negatively affect the hepatic histological injury and apoptosis in the control mice (Fig. 5C).

To confirm the effect of NM on macrophage activation in the liver, double immunofluorescence staining was performed to label the M1-specific antigen F4/80 and iNOS in liver sections [31]. The results demonstrated abundant double-positive macrophages in the livers of septic mice, whereas NM reduced their presence (Fig. S2).

### 3.6 NM *inhibits endothelial ICAM-1 expression and SAA activation in the liver*

**Fig. 6:**
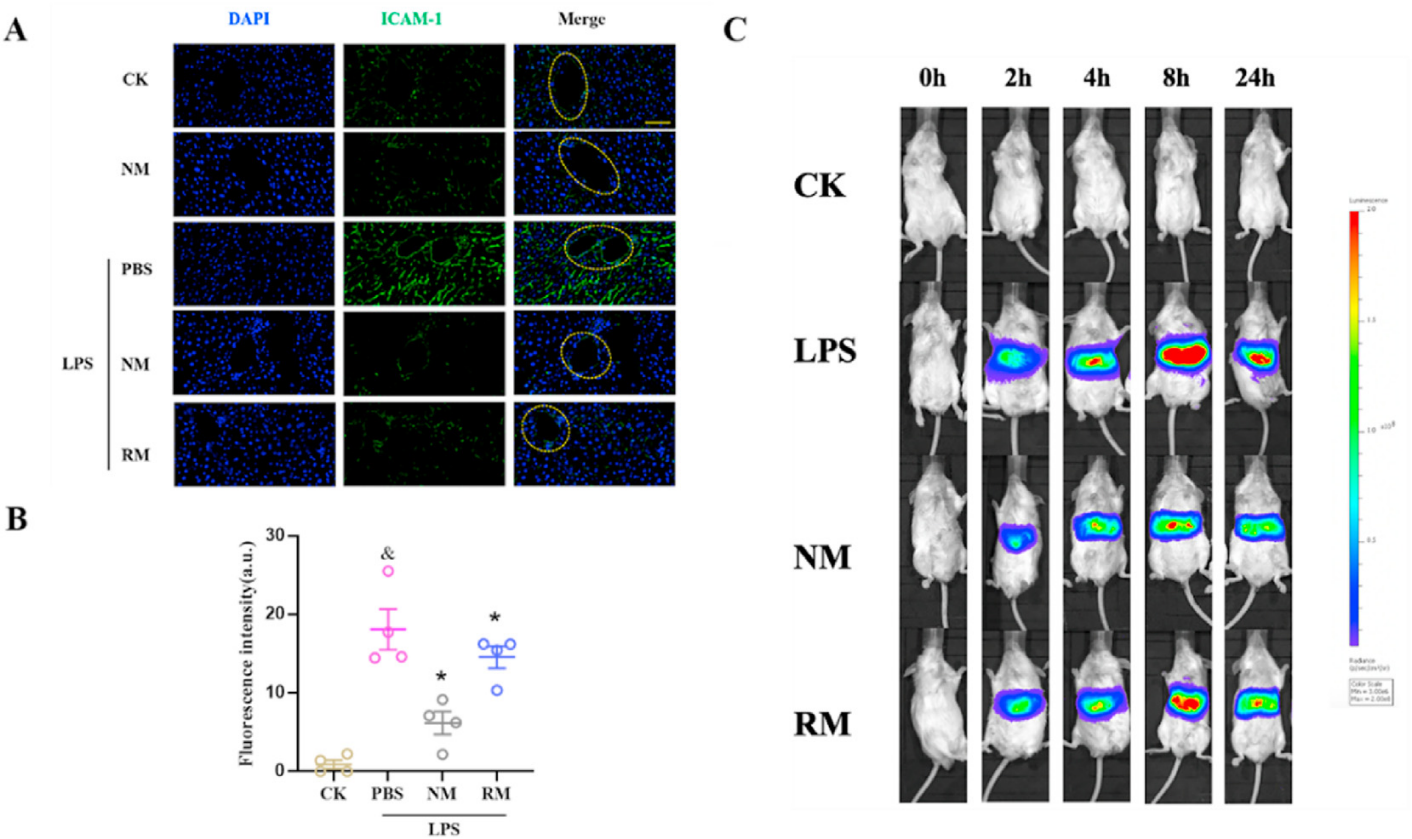
Representative fluorescence images (A) and quantitative analysis (B) show the express levels of intercellular adhesion molecule-1 (ICAM-1) in the liver (Scale bar, 50 ​μm). (C) Dynamic serum Amyloid A (SAA) activation in liver was detected at different time points. ^&^*p* ​< ​0.05 compared with the CK group, ∗*p* ​< ​0.05 compared with the LPS ​+ ​PBS group. Statistical differences between groups were performed by a one-way ANOVA.

Neutrophil infiltration begins with neutrophil-endothelial cell adhesion, which appears to be mediated by adhesion molecules [32]. ICAM-1 on the surface of endothelial cells is key in mediating adhesion and subsequent infiltration of neutrophils. A previous study documented that the selective blockade of ICAM-1 reduces neutrophil infiltration in lungs by 70% [33]. As shown in Fig. 6A and B, the sepsis model mice revealed a significant increase in hepatic ICAM-1 expression compared with the control, while the mice under NM treatment exhibited significant downregulation of LPS-induced endothelial ICAM-1 expression. Notably, there was a continuous and high ICAM-1 expression on the inner surface of the vessel in the sepsis group, and the mice in the NM group showed an intermittent and relatively low ICAM-1 expression. In addition, the mice with RM administration also showed a significant reduction in endothelial ICAM-1 expression compared to the sepsis model mice. These results indicate that NM can alleviate neutrophil infiltration, which was associated with endothelial ICAM-1 expression suppression.

The serum amyloid A (SAA), an acute-phase protein, can be expressed rapidly in response to infections and stress and used as a reliable and sensitive marker of inflammatory states [22,34]. To investigate the impact of NM administration on SAA activation during sepsis, an SAA reporter mouse model was constructed for the non-invasive and dynamic determination of hepatic SAA transcriptional activation. As shown in Fig. 6C, there was no luciferase signal in normal controls. The luciferase signal could be detected 2 ​h after the LPS stimulation, reached its peak at 8 ​h and started to decline at 24 ​h. The mice treated with NM revealed a significant decline in luciferase signal compared to the sepsis model mice. The RM-treated mice exhibited a significant and slightly lower signal than the mice treated with LPS.

### 3.7 NM *directly reduces neutrophil chemotaxis and neutrophil-endothelial cell adhesion in vitro*

**Fig. 7:**
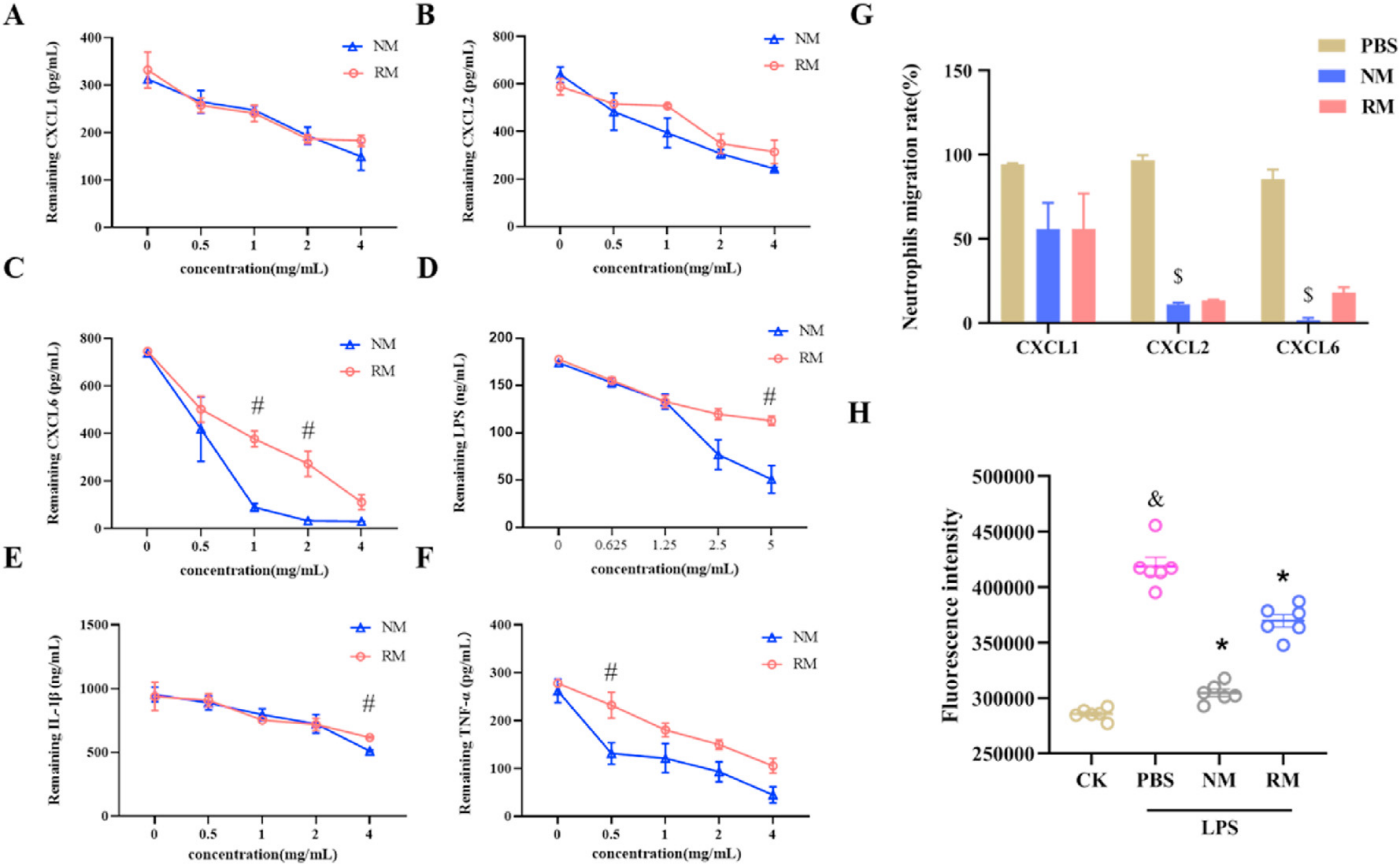
Binding capacity of neutrophil membrane-mimicking nanodecoys (NM) and red cell membrane-mimicking nanovesicles (RM) with CXC-Chemokine Ligand 1 (CXCL1) (A), CXCL2 (B), CXCL6 (C), Lipopolysaccharide (LPS) (D), interleukin (IL)-1β (E), and tumor necrosis factor (TNF)-α (F). (G) The effect of NM on neutrophil chemotaxis. (H) The neutrophil-endothelial cell adhesion indicated by fluorescence intensity. ^&^*p* ​< ​0.05 compared with the CK group, ∗*p* ​< ​0.05 compared with the LPS group, ^#^*p* ​< ​0.05 compared with the NM group, ^$^*p* ​< ​0.05 compared with the PBS group. The unpaired student's t-test was used for the assessment of statistically significant differences between the two groups for Fig. 7C, D, E, and F. Statistical differences between groups were performed by a one-way ANOVA for Fig. 7 G and H. (For interpretation of the references to color in this figure legend, the reader is referred to the Web version of this article.)

To elucidate the potential mechanism by which NM attenuated the sepsis-induced acute liver injury, the effect of NM on neutrophil chemotaxis *in vitro* was assessed. In the setting of sepsis, the locally produced chemokines, such as CXCL1, CXCL2, and CXCL6, could lead to neutrophil chemotaxis and subsequent neutrophil infiltration by ligand-receptor binding [35,36]. Systematic neutralization of chemokines reduced the severity of inflammation by blocking neutrophil chemotaxis [37]. Theoretically, NM retains the chemokines-binding properties, thus, the binding ability of NM to sequester chemokines, including CXCL1, CXCL2, and CXCL6, was first investigated. As shown in Fig. 7A and B, the NM and RM showed the similar binding ability at various concentrations for CXCL1 and CXCL2 in a dose-dependent manner. For CXCL6, although both NM and RM exhibited enhanced binding ability with increased concentrations, NM exerted more powerful binding ability than RM at the concentrations of 1 ​mg/mL and 2 ​mg/mL, especially NM and RM could neutralize 649.7 ​pg and 368.8 ​pg CXCL6 at a concentration of 1 ​mg/mL, respectively (Fig. 6C).

In the development of sepsis, inflammatory mediators can trigger and amplify acute liver injury by directly inducing hepatocyte injury. Thus, the binding ability of NM to sequester inflammatory mediators, including LPS, IL-1β, and TNF-α, was investigated. As shown in Fig. 7D, E, F, and S3, NM and RM exhibited the augmenting binding abilities with LPS, IL-1β, TNF-α, and SAA in a dose-dependent manner, and NM had stronger bonding ability with LPS than RM at a concentration of 5 ​mg/mL. For IL-1β, NM and RM revealed comparable bonding abilities at the concentrations of 0.5, 1, and 2 ​mg/mL, and NM had stronger bonding ability with IL-1β than RM at a concentration of 4 ​mg/mL. For TNF-α, NM showed a stronger bonding ability with TNF-α than RM at a concentration of 0.5 ​mg/mL. In addition, NM showed a stronger bonding ability with SAA than RM at a concentration of 2 ​mg/mL (Fig. S3).

Neutrophil chemotaxis towards chemokines was evaluated by a transwell assay. As shown in Fig. 7G, NM and RM showed suppressive impact on neutrophil chemotaxis towards CXCL1 but without statistically significant. NM significantly inhibited neutrophil chemotaxis towards CXCL2 by 89.0%, and RM showed suppressive impact on neutrophil chemotaxis towards CXCL2, however, without statistically significant. NM markedly inhibited neutrophil chemotaxis towards CXCL6 by 98.3%, and RM showed suppressive impact on neutrophil chemotaxis towards CXCL6 but without statistically significant.

As shown in Fig. 7H, there was a significant increment in adhesion of neutrophils on activated endothelial cells after LPS exposure, and NM and RM significantly decreased the number of adherent neutrophils compared with the LPS-treated cells. In addition, the NM exhibited a significant suppression of neutrophil adhesion compared with RM. These results are consistent with the confocal imaging of neutrophil-endothelial cell adhesion (Fig. S4).

### 3.8 NM *reduces LPS-induced hepatocyte injury by neutralizing inflammatory mediators*

**Fig. 8:**
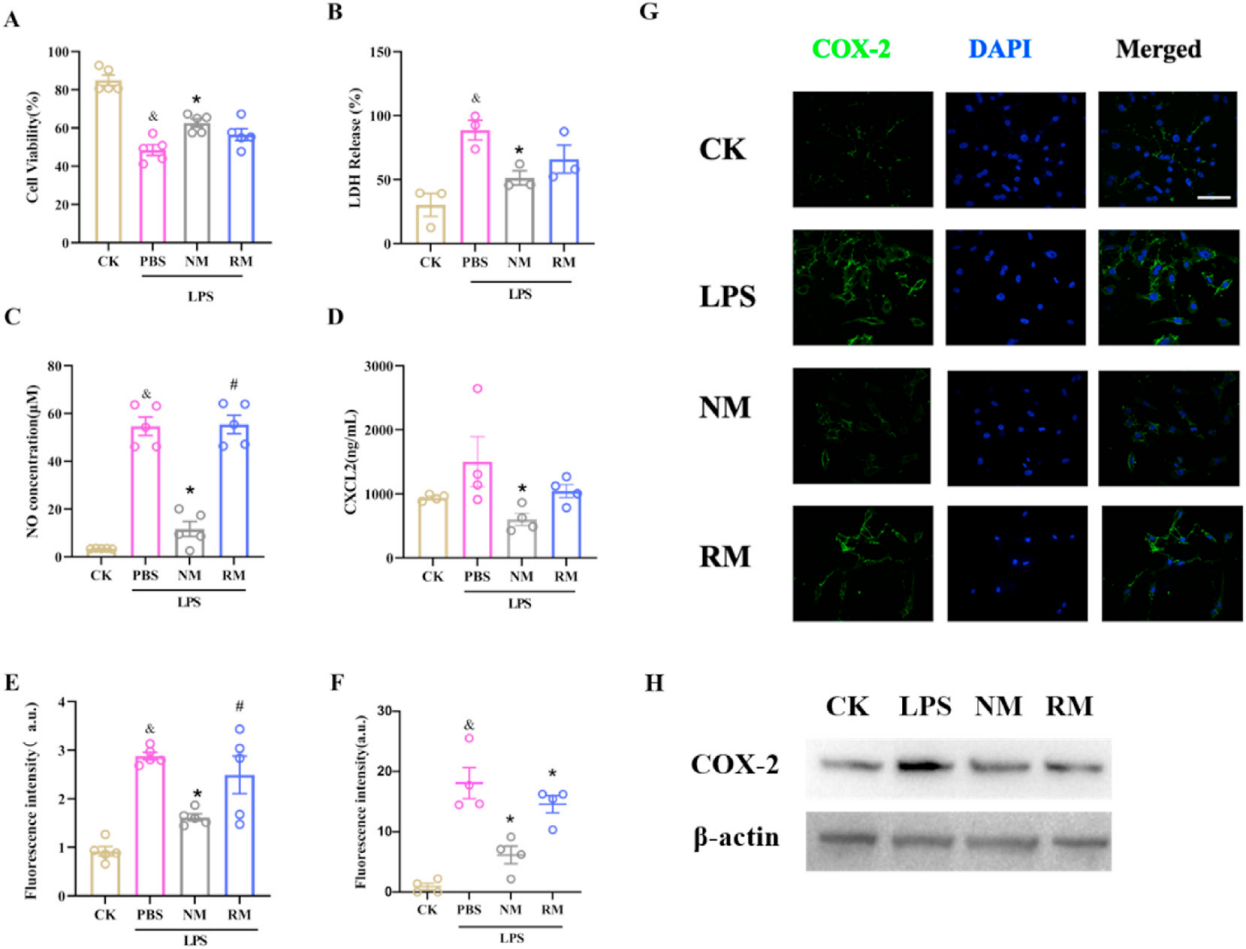
Effects of neutrophil membrane-mimicking nanodecoys (NM) and red cell membrane-mimicking nanovesicles (RM) on the LPS-induced cytotoxicity (A), lactate dehydrogenase (LDH) release (B), nitric oxide (NO) secretion (C), CXC-Chemokine Ligand 2 (CXCL2) expression (D), Reactive Oxygen Species (ROS) generation indicated by fluorescence intensity (E), and Cyclooxygenase-2 (COX-2) expression (Fig. 8F–H) in NCTC1469 murine liver cells; Scale bars, 50 ​μm; ^&^*p* ​< ​0.05 compared with the CK group; ∗*p* ​< ​0.05 compared with the LPS ​+ ​PBS group; ^#^*p* ​< ​0.05 compared with the LPS ​+ ​NM group. Statistical differences between groups were performed by a one-way ANOVA for Fig. 8A, B, C, E, and F. Statistical differences between groups were performed by SNK multiple range test for Fig. 8D. (For interpretation of the references to color in this figure legend, the reader is referred to the Web version of this article.)

The effect of NM on LPS-induced hepatocyte injury *in vitro* was evaluated. Cell viability analysis indicated that the cell viability was significantly decreased after LPS stimulation, and treatment with NM significantly elevated the cell viability compared with LPS-stimulated cells (Fig. 8A). RM showed a tendency to increase the cell activity of LPS-stimulated cells, but there was no statistical difference. LPS stimulation also dramatically increased LDH release and NO secretion, which were greatly reversed by the NM treatment (Fig. 8B and C). It further showed that LPS could increase the expression level of iNOS, which is pointed out as the major source of NO during sepsis [38], and RM and NM could reduce level of iNOS (Fig. S5).

Treatment with RM did not significantly decrease LDH release compared to the LPS-stimulated cells. In addition, there was a non-significant increase in LPS-treated cells compared with control cells for CXCL2 secretion, while NM treatment significantly suppressed the LPS-induced CXCL2 secretion (Fig. 8D), while the RM treatment exhibited non-significant inhibition of LPS-induced CXCL2 secretion.

Previous studies have demonstrated that ROS generation induced by LPS stimulation positively regulates COX-2 expression, which contributes to inflammatory reaction [39,40]. As indicated in Fig. 8E, LPS stimulation significantly elevated the ROS level in hepatocytes, which was largely diminished by NM treatment. The RM treatment showed non-significant inhibition on ROS generation compared to the LPS-stimulated cells. The results of immunofluorescence staining and western blot indicated LPS exposure upregulated the COX-2 expression, which was prevented by NM treatment (Fig. 8F–H). The RM treatment showed a weak suppression of COX-2 expression.

## 4 Discussion

**Fig. 9:**
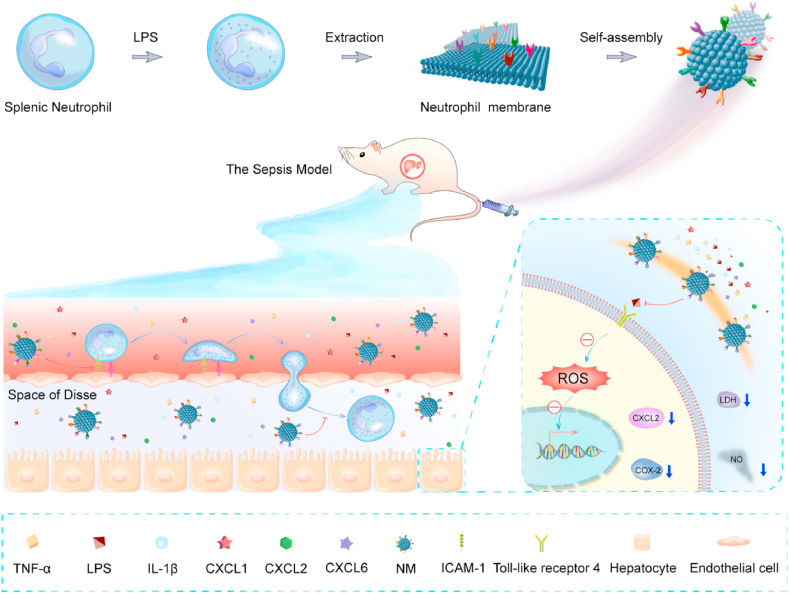
Schematic illustration of the NM fabrication and therapeutic effect for sepsis-induced acute liver injury.

In the current study, we demonstrated that NM can neutralize the neutrophil chemokine and inflammatory mediators, mitigate neutrophil chemotaxis and adhesion, and reduce inflammatory mediators-induced hepatocyte injury *in vitro*. Further *in vivo* studies confirm that treatment with NM displays good biocompatibility and reduces neutrophil infiltration, and liver apoptosis, and subsequently provide a robust superiority in improving sepsis-induced acute liver injury and mortality (Fig. 9).

Our study indicated that the neutralization of NM with endotoxin and proinflammatory cytokines appeared to be one of the important mechanisms underlying therapeutic efficacy. As NM retains the antigenic surface of the neutrophils, we believe that NM has a unique advantage in regulating neutrophil behavior. Herein, we noticed that NM could provide a therapeutic efficacy on sepsis by directly regulating neutrophil behaviors, such as chemotaxis and adhesion, which were important novel mechanisms for the therapeutic effect of NM in sepsis. In addition, Annexin 1 might also play an important role in the therapeutic effect of NM. Previous studies have demonstrated that Annexin 1, which could be detected on the neutrophil cell membrane [41], exhibit anti-inflammatory effects in several disease models [42,43]. We assumed that Annexin 1 in NM could partly contribute to the therapeutic efficacy of NM in sepsis.

NM and RM were simultaneously evaluated in this study. NM revealed an enhanced therapeutic advantage over RM in our observations. RM only showed weak therapeutic efficacy in sepsis in general. The differences in therapeutic efficacy might be related to the role of original cells in inflammation. Neutrophils are sensitive to the changes of the immune microenvironment and play pivotal roles in inflammatory reactions during sepsis [44]. Thus, NM is likely to have more types and higher density of binding sites for chemokines and inflammatory cytokines and subsequent stronger regulation ability towards neutrophils compared with RM. Our study also found that NM exhibits more powerful neutralization for LPS, TNF-α, and CXCL6, which might partly explain the therapeutic advantage of NM over RM in sepsis treatment. Nevertheless, although our study provided some important evidence, the precise mechanism regarding the therapeutic advantage of NM over RM requires further investigation.

Our study indicated that NM is an effective immunomodulator and detoxicant for multiple inflammatory mediators and could exhibit a powerful therapeutic effect for sepsis. Similar to sepsis, we speculate the NM can also be adapted to treat other inflammatory-associated diseases, including inflammatory bowel disease and periodontitis, by modulating neutrophil behavior and reducing the inflammatory response in the disease process. Although NM has shown great therapeutic potential for inflammatory-associated diseases, it should be comprehensively investigated before clinical applications. It is critical to consider when to use NM for optimum efficacy. The hyperinflammatory response is the main reason for deaths during the early stage of sepsis [45]. We believe that NM could exhibit the best therapeutic effect in sepsis by preventing early excessive inflammation and late immunosuppression when administrated as early as possible. NM might not be a suitable treatment once patients with sepsis have dysfunctional immune responses due to immunosuppression.

Owing to the limited blood volume in mice and biological replicate needs, we conducted four animal experiments for the plasma assays. The plasma for AST and ALT measurements was obtained from the same experiment; the plasma for the DBIL measurement was obtained from another experiment; the plasma for ALB/GLB measurement was obtained from another experiment; and the plasma samples for cytokine measurements were obtained from yet another experiment. Further, the liver samples for MDA content measurement, MPO activity measurement, and immunostaining experiments were obtained from the same experiment. Hepatic mRNA expression level determination, lung MDA content measurement, and BUN measurement were conducted using samples from the same experiment.

The observation time in this study is mainly based on our previous research. we observed notable acute liver injury in mice 6-12 ​h after the LPS injection, and the therapeutic effect of treatment drugs can also be clearly shown [20,46]. In addition, other studies also measured liver injury at 6-12 ​h [47,48]. In addition, there were hyperemia in pathological sections of NM group and RM group, this phenomenon may be related to the fact that the animal did not bleed before liver tissue collection. Further, we believe that the short observation time is an important reason why there is not much neutrophil infiltration in the liver of the model group in HE staining.

In this study, the protection of NM on lung and kidney also was initially studied, we found the NM treatment also can significantly reduce the lung MDA content and blood urea nitrogen (BUN) content induced by LPS administration (Fig. S6). These results suggest that NM also exhibit protective effect on lung and kidney, which needs further research.

## 5 Conclusions

In summary, this study developed a simple method to construct neutrophil membrane-derived nanovesicles and further demonstrated their therapeutical effect for sepsis-induced liver injury. The detailed evaluations indicate that NM exerts excellent biocompatibility and prominent protective effects for sepsis-induced acute liver injury and mortality, which might be mediated by directly regulating neutrophil chemotaxis and adhesion as well as reducing inflammatory mediators-induced hepatocyte injury. These findings imply a promising therapeutic intervention strategy for the treatment of sepsis-induced acute liver injury or other inflammation-related diseases.

## Authorship contributions

Yao Xiao: Methodology, Investigation, Formal analysis, Data curation, Writing – original draft. Chao Ren: Methodology, Formal analysis, Data curation, Conceptualization. Gan Chen: Formal analysis, Conceptualization, Data curation, Supervision, Writing – original draft, Writing – review & editing. Pan Shang: Formal analysis, Data curation. Xiang Song: Methodology, Software, Validation, Visualization. Guoxing You: Methodology, Software, Formal analysis, Data curation. Shaoduo Yan: Methodology, Investigation, Resources, Validation. Yongming Yao: Supervision, Conceptualization, Project administration, Writing – review & editing. Hong Zhou: Funding acquisition, Project administration, Supervision, Conceptualization, Writing – review & editing.

## Funding

This work was supported by 10.13039/501100001809National Natural Science Foundation of China (82170229) and Foundation Strengthening Program Technology Fund Project (2019-JCJQ-JJ-164).

## Data availability

The data that support the findings of this study are available from the corresponding authors upon reasonable request.

## Declaration of competing interest

The authors declare that they have no known competing financial interests or personal relationships that could have appeared to influence the work reported in this paper.
