# Supplementary material for: Neutrophil membrane-mimicking nanodecoys with intrinsic anti-inflammatory properties alleviate sepsis-induced acute liver injury and lethality in a mouse endotoxemia model
Source: Mater Today Bio. 2022 Mar 16;14:100244. doi: 10.1016/j.mtbio.2022.100244 (PMC8956822; doi:10.1016/j.mtbio.2022.100244)
Supplement: Multimedia component 1 [file mmc1.docx]

**Neutrophil membrane-mimicking nanodecoys with intrinsic anti-inflammatory properties alleviate sepsis-induced acute liver injury and lethality in a mouse endotoxemia model**

Yao Xiao^1†^, Chao Ren^2,3†^, Gan Chen^1†*^, Pan Shang^1^, Xiang Song^4^, Guoxing You^1^, Shaoduo Yan^1^, Yongming Yao^2*^, Hong Zhou^1*^

^1^Institute of Health Service and Transfusion Medicine, Beijing, China

^2^Translational Medicine Research Center, Fourth Medical Center and Medical Innovation Research Division of the Chinese PLA General Hospital, Beijing, China

^3^Department of Pulmonary and Critical Care Medicine, Beijing Chaoyang Hospital, Capital Medical University, Beijing, China

^4^Beijing Institute of Radiation Medicine, Beijing, China

| **Gene** | **Forward primer** | **Reverse primer** |
| --- | --- | --- |
| IL-6 | TTCCATCCAGTTGCCTTCTT | TTCTGCAAGTGCATCATCGT |
| IL-1β | GGACAGAATATCAACCAACAAGTGATA | GTGTGCCGTCTTTCATTACACAG |
| TNF-α | AAGCCTGTAGCCCACGTCGTA | GGCACCACTAGTTGGTTGTCTTTG |
| 18s | GTAACCCGTTGAACCCCATT | CCATCCAATCGGTAGTAGCG |
| *18s was used as the internal reference for the expression of IL-6, IL-1β, and TNF-α. | | |

Table S1. Primer information for genes in this study.


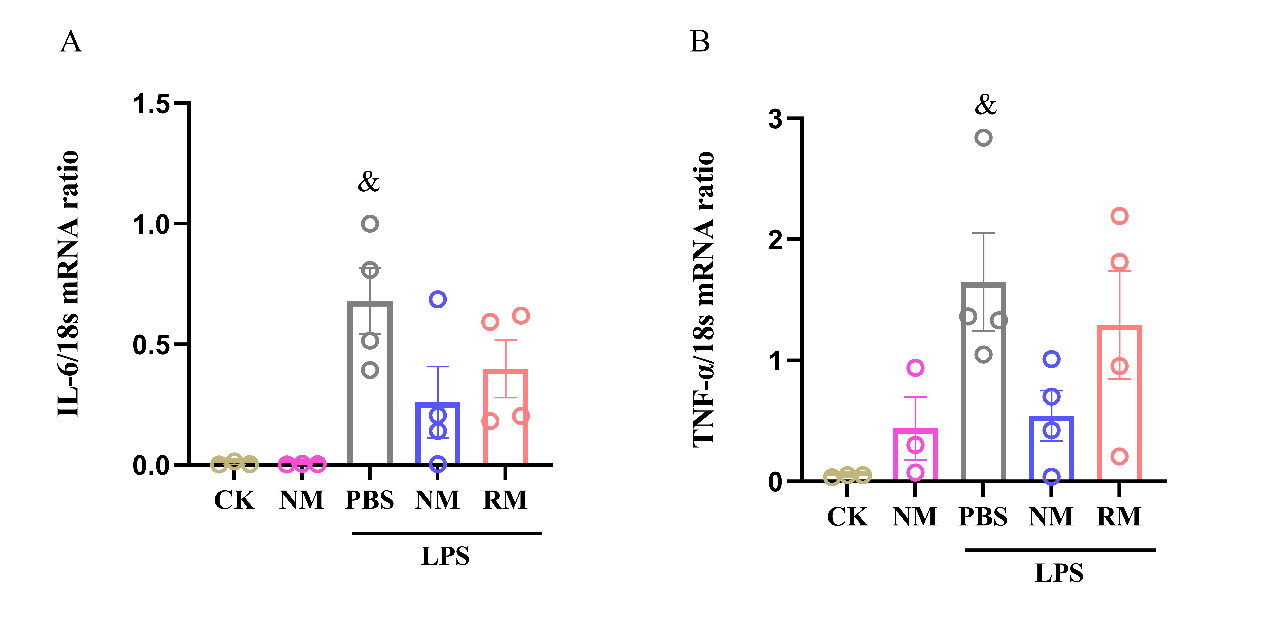


Fig. S1. IL-6 (A) and TNF-α (B) mRNA levels in livers were analyzed by real-time PCR. ^&^*p* < 0.05 compared with the CK group. Statistical differences between groups were performed by a one-way ANOVA for Fig. S1A. Statistical differences between groups were performed by SNK multiple range test for Fig. S1B.


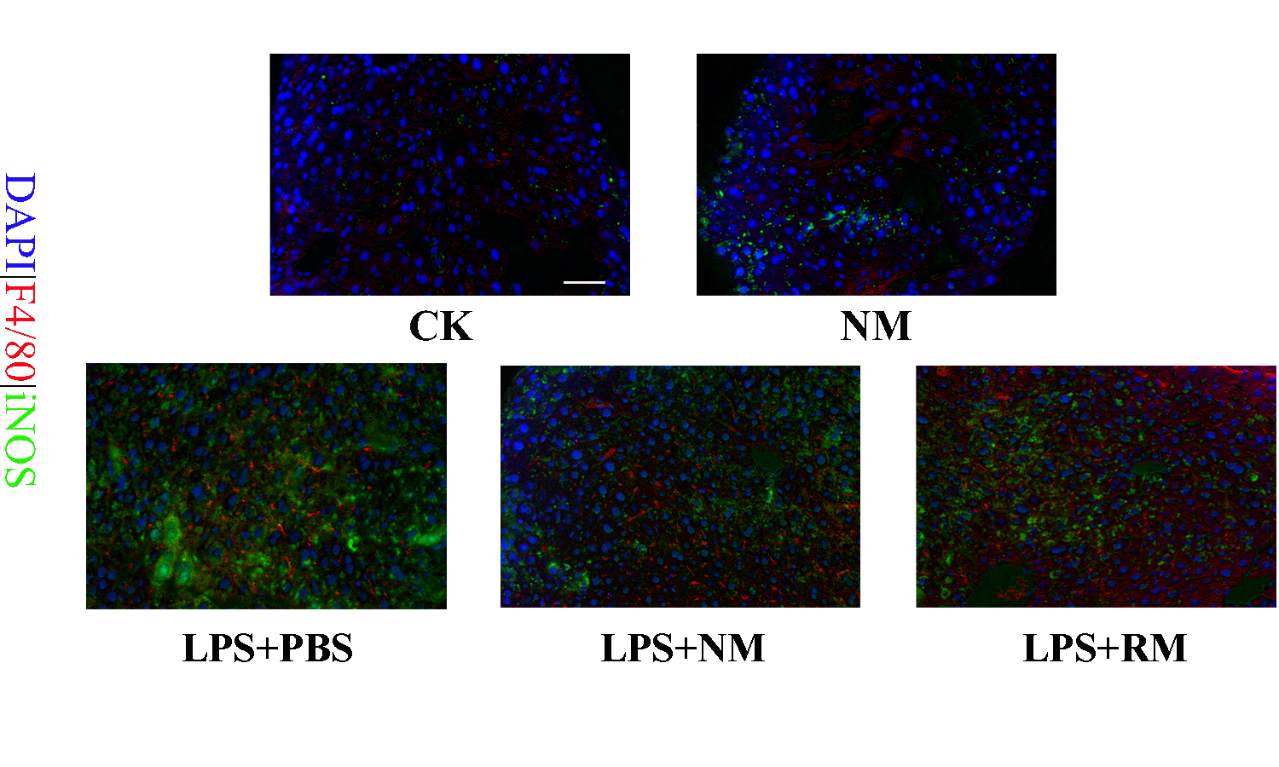


Fig. S2. Representative fluorescence images show the hepatic expression levels of F4/80 and inducible nitric oxide synthase (iNOS) (Scale bar, 50 μm).


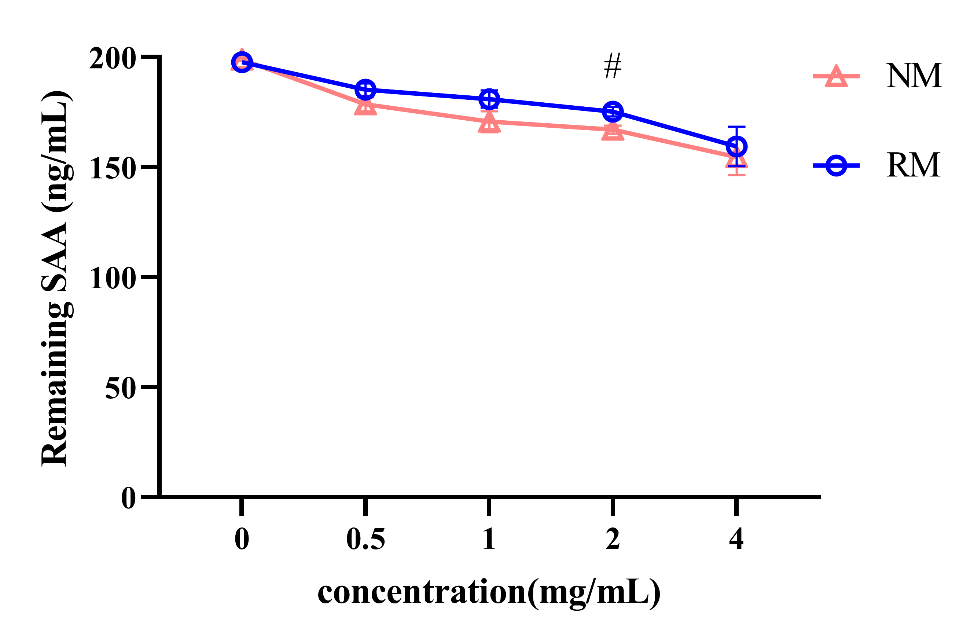


Fig. S3. Binding capacity of neutrophil membrane-mimicking nanodecoys (NM) and red cell membrane-mimicking nanovesicles (RM) with serum Amyloid A (SAA). ^#^*p* < 0.05 compared with the NM group. The unpaired student’s t-test was used for the assessment of statistically significant differences between the two groups for Fig. S3.


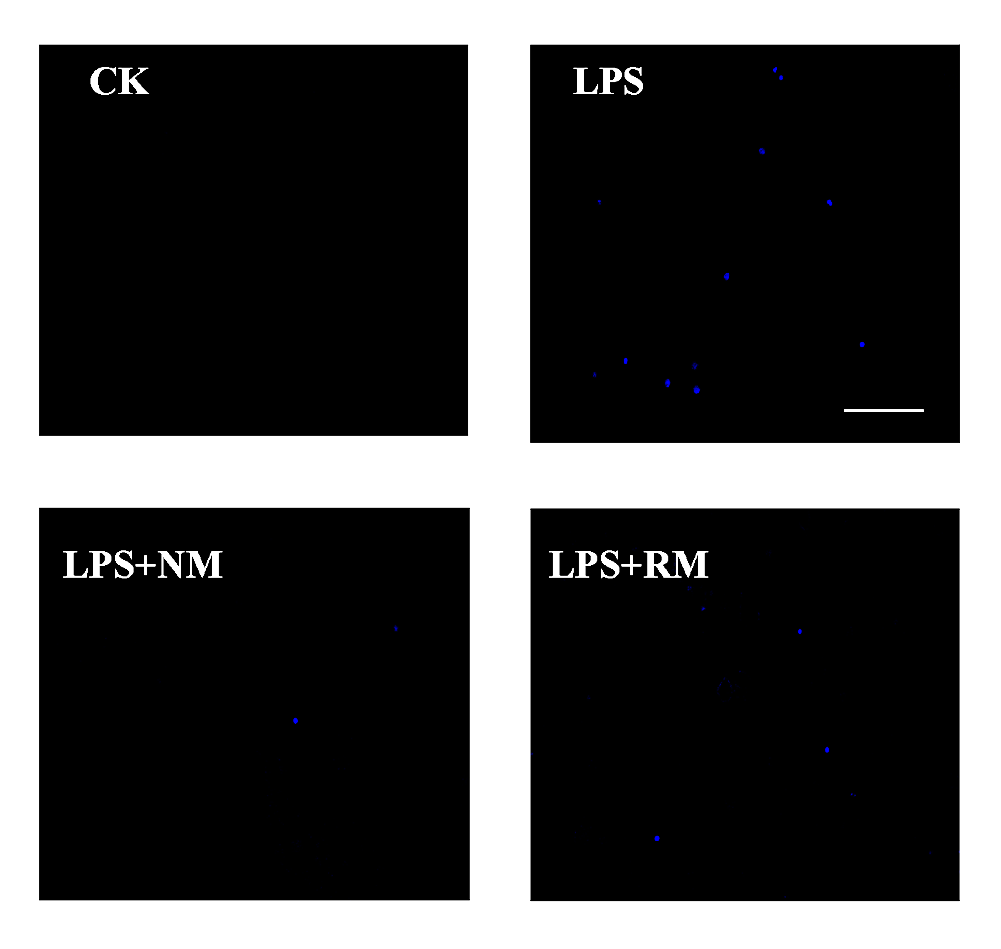


Fig. S4. Confocal imaging of neutrophil-endothelial cell adhesion (Scale bar, 200 μm)


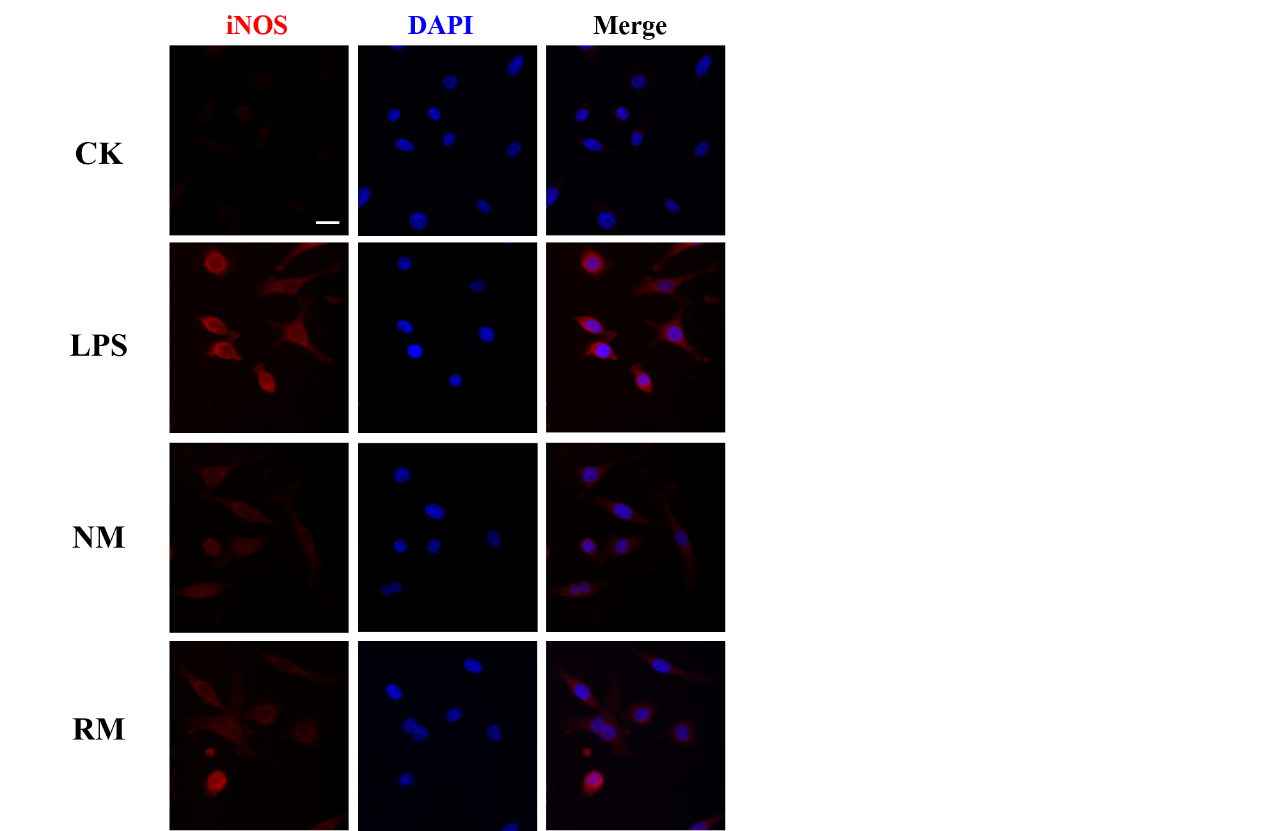


Fig. S5. Immunofluorescence images of inducible nitric oxide synthase (iNOS) expression in NCTC1469 cells (scale bar, 10 μm)


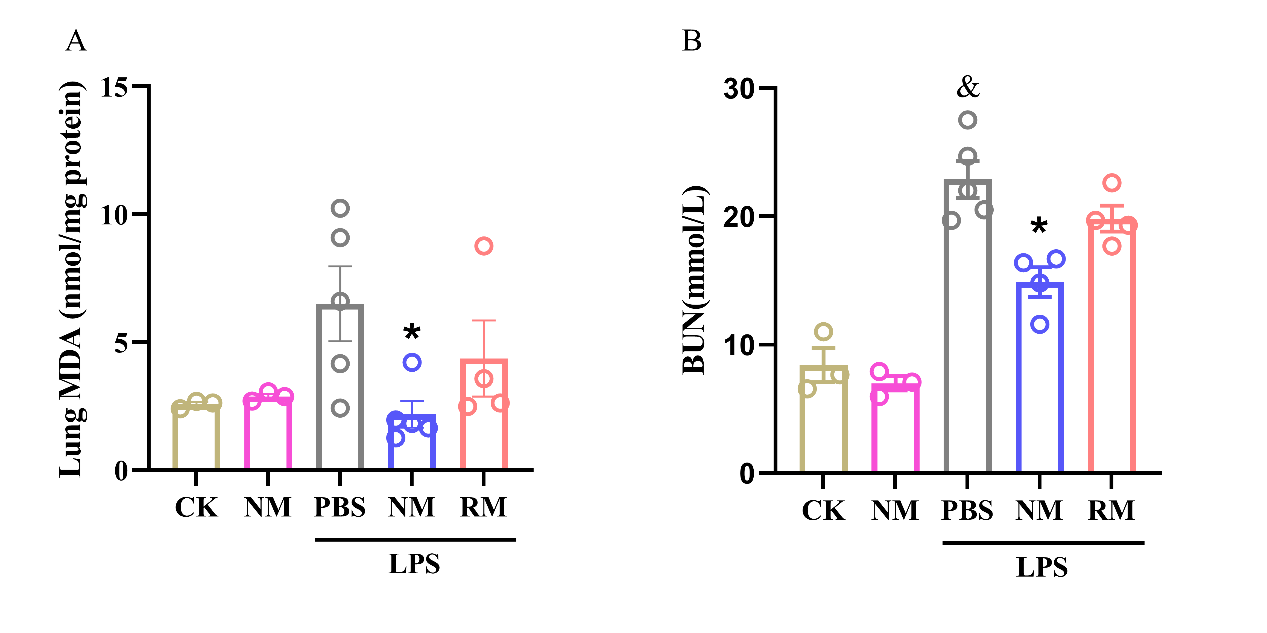


Fig. S6. Lung malondialdehyde (MDA) content (A) and the level of blood urea nitrogen (BUN) in the serum (B). ^&^*p* < 0.05 compared with the CK group, ^&^*p* < 0.05 compared with the CK group. Statistical differences between groups were performed by a one-way ANOVA.
